# Bichloride of Methylene

**Published:** 1868-02

**Authors:** 


### ARTICLE IV.

### *Bichloride of Methylene.*

Dr. B. W. Richardson, so well and favorably known by his researches upon anæsthesia, and his additions to our

*armamentarium* in this direction has recently introduced a new anæsthetic, to which we propose to devote a little attention.

Bichloride of methylene belongs to the monocarbon group of organic compounds ; that is to say, its radical is one of those, into the composition of which a single atom of carbon enters. If we take for example, *marsh gas ;* which is a tetrahydride of carbon, and which may be written $CH_4$, we shall find that we can express its composition by the formula, CH, $H_3$, which indicates that it may be considered a hydride of methyl, $CH_3$ being the expression for that radical. A very good reason for this opinion is found in the reactions of chlorine with marsh gas. Chlorine may substitute the hydrogen, to form the chloride of methyle, $CH_3Cl$. The action may go further than this, and two equivalents of chlorine may substitute two equivalents of hydrogen, when the radical will be decomposed to methylene, $CH_2$, and we shall have the formula $CH_2Cl_2$, or bichloride of methylene, the substance under consideration. Nor need the action stop here. The last-named radical may be still further reduced to CH, or formyl, by the substitution of three atoms of hydrogen by three of chlorine, and this will give us $CHCl_3$, terchloride of formyl or chloroform. Indeed the whole of the hydrogen may be substituted by chlorine, and then we have $CCl_4$, tetrachloride of carbon.

It is remarkable that every one of these substances possesses anæsthetic powers. That our readers may see their relations at a glance, a table is subjoined.

| | |
|---|---|
| $C\ H_2\ H$ ; | Marsh Gas (Hydride of Methyl.) |
| $C\ H_3\ Cl$ ; | Chloride of Methyl. |
| $C\ H_2\ Cl_2$ ; | Bichloride of Methylene. |
| $C\ H\ Cl_3$ ; | Chloroform. |
| $C\ C_4l$; | Tetrachloride of Carbon. |

To complete the view of this series, there may be added the formula $\left.\begin{matrix} C\ H_3 \\ H \end{matrix}\right\} 0$—Methylic Alcohol—Wood Spirit or Naphtha.

It might be supposed that the whole series possessed anæsthetic properties in various degrees. Dr. Richardson, however, experimentally determined that pigeons, birds known to be extremely sensitive to anæsthetics, could live in an atmosphere containing thirty-five per cent. of hydride of methyl, marsh gas, or fire damp (for it goes under all these aliases) for half an hour, and that he himself could breathe with impunity the air coming from their chamber. When death is produced, it occurs in a gentle sleep. The lungs, heart and blood retain their natural conditions, and resuscitation is easily accomplished. Of the anæsthetic action of the other members of the series, we shall speak after we have paid some attention to the main subject of our remarks.

Bichloride of methylene is prepared by the action of sulphuric acid on zinc in chloroform, the nascent hydrogen substituting an equivalent of chlorine which is converted into hydrochloric acid. Thus:

$$C\,H\,Cl_3 + 2\,H = C\,H_2\,Cl_2 + H\,Cl.$$

It is a colourless liquid, with an odour resembling that of chloroform. Its boiling point is 88° F., its specific gravity 1.344, its vapour density 2.937. (Regnault makes it 3.012.) The following table exhibits its relation to its congeners, at a glance.

| | Boiling Point. F. | Spec. Grav. Water=1. | Dens. Vap Air=1. |
|---|---|---|---|
| Chloride of Methyl, | 6° | | 1.745 |
| Bichloride of Methylene, | 88° | 1.344 | 2.937 |
| Chloroform, | 142° | 1.495 | 4.122 |
| Tetrachloride of Carbon, | 172° | 1.599 | 5.321 |
| Ether ($C_2\,H_5O$) | 92° | 0.720 | 1.547 |
| Amylene ($C_5\,H_{10}$) | 95° | 0.659 | 2.419 |

It will be seen that it boils at a lower temperature than any of these liquids, not excepting ether. The boiling point of this latter, however, so nearly approximates that of the new anæsthetic that they can be mixed together and be vaporized equally, thus obviating an objection to which the mixture of chloroform and ether is liable. It is also miscible with chloroform in all proportions.

The establishment of anæsthesia by this agent is not so speedy as by chloroform. The subject yields gradually to its influence, the most marked peculiarity of its action being the absence of Snow's second stage of anæsthesia, or that of excitement. It enters readily into the circulation, and keeps up the insensibility so well that it does not require to be frequently re-administered. Herein, it differs from ether and amylene which produce a much briefer narcotism. In pigeons it occasionally produces vomiting. Whether this unpleasant circumstance will attend its action upon the human subject, experience must determine. The recovery is quiet and complete.

It should be administered upon a sponge, in a balloon or Clover's bag, or from a funnel. Snow's inhaler presents too small a surface for evaporation. In the earlier stages of administration, a little more of this substance should be given than of chloroform, as it is more easily vaporized and the loss is greater. One drachm of bichloride of methylene corresponds to forty grains of chloroform.

When the action of the remedy is pushed to a fatal issue, Dr. Richardson informs us that the resistance to death is as fourteen to five in favour of bichloride of methylene against tetra-chloride of carbon, and as fourteen to nine against chloroform. Equally marked differences in the action of these anæsthetics on muscular irritability have been observed. Tetrachloride of carbon extinguishes this vital activity of muscles in seven minutes, chloroform in twenty-three minutes, and bichloride of methylene in fifty-eight minutes. Dr. Richardson has also observed a difference in the action of these different chlorides upon the lungs and heart. Chloroform leaves the lungs bloodless, the right cavities of the heart engorged, aad the left cavities empty. Carbonic acid, and occasionally in a less degree, ether and tetrachloride of carbon, leave the lungs congested and both sides of the heart containing blood.

Where death occurs from bichloride of methylene, the lungs and both sides of the heart contain blood, but they are not unduly distended.

The energy of these anæsthetics would seem to be proportioned to the amount of chlorine they contain. Thus, chloride of methyl produces sleep, and can, if used in sufficient quantities, destroy life. Muscular irritability remains an hour after death. Dissolved in water and drunk it produces intoxication, which is, however, of short duration. The most dangerous of these agents is believed to be tetrachloride of carbon, which certainly contains the largest proportion of chlorine. The remarks already made show the relation of the other members of the series.

Several cases have been reported in which the new anæsthetic has been employed in severe surgical operations on the human subject. In all the most satisfactory anæsthesia has been obtained, and in none have any untoward symptoms been observed.

---

